# Neutrophil Elastase Subverts the Immune Response by Cleaving Toll-Like Receptors and Cytokines in Pneumococcal Pneumonia

**DOI:** 10.3389/fimmu.2018.00732

**Published:** 2018-04-25

**Authors:** Hisanori Domon, Kosuke Nagai, Tomoki Maekawa, Masataka Oda, Daisuke Yonezawa, Wataru Takeda, Takumi Hiyoshi, Hikaru Tamura, Masaya Yamaguchi, Shigetada Kawabata, Yutaka Terao

**Affiliations:** ^1^Division of Microbiology and Infectious Diseases, Niigata University Graduate School of Medical and Dental Sciences, Niigata, Japan; ^2^Research Center for Advanced Oral Science, Niigata University Graduate School of Medical and Dental Sciences, Niigata, Japan; ^3^Division of Periodontology, Niigata University Graduate School of Medical and Dental Sciences, Niigata, Japan; ^4^Department of Microbiology and Infection Control Sciences, Kyoto Pharmaceutical University, Kyoto, Japan; ^5^Division of Oral Science for Health Promotion, Niigata University Graduate School of Medical and Dental Sciences, Niigata, Japan; ^6^Faculty of Dentistry, Niigata University, Niigata, Japan; ^7^Department of Oral and Molecular Microbiology, Osaka University, Graduate School of Dentistry, Osaka, Japan

**Keywords:** neutrophil elastase, pneumonia, innate immune response, toll-like receptor, *Streptococcus pneumoniae*, cytokines

## Abstract

Excessive activation of neutrophils results in the release of neutrophil elastase (NE), which leads to lung injury in severe pneumonia. Previously, we demonstrated a novel immune subversion mechanism involving microbial exploitation of this NE ability, which eventually promotes disruption of the pulmonary epithelial barrier. In the present study, we investigated the effect of NE on host innate immune response. THP-1-derived macrophages were stimulated with heat-killed *Streptococcus pneumoniae* or lipopolysaccharide in the presence or absence of NE followed by analysis of toll-like receptor (TLR) and cytokine expression. Additionally, the biological significance of NE was confirmed in an *in vivo* mouse intratracheal infection model. NE downregulated the gene transcription of multiple cytokines in THP-1-derived macrophages through the cleavage of TLRs and myeloid differentiation factor 2. Additionally, NE cleaved inflammatory cytokines and chemokines. In a mouse model of intratracheal pneumococcal challenge, administration of an NE inhibitor significantly increased proinflammatory cytokine levels in bronchoalveolar lavage fluid, enhanced bacterial clearance, and improved survival rates. Our work indicates that NE subverts the innate immune response and that inhibition of this enzyme may constitute a novel therapeutic option for the treatment of pneumococcal pneumonia.

## Introduction

Bacterial pneumonia constitutes a leading cause of morbidity and mortality worldwide, being responsible for approximately 3.5 million deaths annually (1). Among all bacteria, *Streptococcus pneumoniae* represents the most common cause of pneumonia in all age groups. Although antibiotics comprise the primary treatment of choice for pneumonia, antimicrobial resistance among *S. pneumoniae* has increased significantly in past decades. Additionally, the total costs of pneumococcal pneumonia reach $2.5 billion per year and are predicted to increase with the growth in the aging population in the United States (2). Therefore, basic research is essential to provide novel therapeutic targets for pneumococcal pneumonia.

Neutrophil elastase (NE) is a serine protease that degrades outer membrane proteins localized on the surface of Gram-negative bacteria and exerts antimicrobial activity (3). Although NE is required for host defense against a wide variety of bacteria, NE also degrades host extracellular-matrix proteins as well as epithelial cadherin, which is a cell–cell adhesion molecule with pivotal roles in epithelial cell behavior and tissue formation, and causes lung epithelial disruption (4, 5). Additionally, NE cleaves various host proteins, such as lung-surfactant proteins (6), vascular endothelial growth factor (7), C1 inhibitor (8), and C5a receptor (9), modulates inflammation, and promotes tissue remodeling. Generally, the proteolytic activity of NE is regulated by α1-antitrypsin, an endogenous NE inhibitor. However, during an acute inflammatory response, macrophages and neutrophils release a variety of proteases, including NE, proteinase 3, matrix metalloproteinases (MMPs), and cathepsins (10). Among these, MMPs, particularly MMP-12, degrade and inactivate α1-antitrypsin, thereby enhancing the activity of NE and causing tissue injury (11). In this regard, it has been reported that NE level is increased in bronchoalveolar lavage fluid (BALF) from patients with severe pneumonia (12). In animal models, intratracheal *S. pneumoniae* infection causes acute lung injury characterized by an increase in neutrophil accumulation and NE activity in BALF (13, 14). Together, these findings indicate that excessive release of NE from neutrophils can damage surrounding tissues and contribute to the lung dysfunction associated with pneumonia.

In order to reduce the excess inflammatory response and lung injury, various inhibitors of NE have been tested in various lung diseases, including severe pneumonia (15). Studies evaluating the effect of NE in the animal model of *Pseudomonas aeruginosa*-induced pneumonia have reported conflicting results. Cantin and Woods reported that the NE inhibitor significantly decreased NE activity and enhanced clearance of bacteria in the lungs (16), whereas Honoré et al. did not obtain positive results (17). In the animal model of pneumococcal pneumonia, NE inhibitor enhanced bacterial clearance and delayed mortality (13, 14). Although there are few descriptive studies of NE inhibitors in humans with bacterial pneumonia, a previous study suggested that early administration of NE inhibitor may improve acute lung injury and survival rate in severe pneumonia (18). Taken together, these findings suggest that not only bacterial pathogens but also NE contribute, at least in part, to the pathological progression of bacterial pneumonia.

We have previously reported that pneumolysin, a pneumococcal pore-forming toxin, induced cell lysis in human neutrophils, leading to the release of NE (19). Subsequently, NE induced the detachment of alveolar epithelial cells, leading to the disruption of pulmonary defenses. NE also impaired phagocytic activity in macrophages *in vitro*. We have also suggested the possibility that NE decreases interleukin (IL)-8 level in the culture supernatant from human alveolar epithelial cells (19). These data suggest that *S. pneumoniae* exploits NE leakage from neutrophils to subvert host innate immune responses. However, the effects of NE on innate immune responses are not fully understood. The main objective of this study was, therefore, to understand how NE inhibits cytokine production from innate immune cells and to determine its roles in a mouse model of pneumococcal pneumonia. Our data presented here demonstrate the immune subversion mechanism of NE, which caused development of pneumococcal bacteremia in experimental pneumonia. Our study thus provides a novel host modulation therapy for pneumonia utilizing an NE-specific inhibitor.

## Materials and Methods

### Mice and Bacteria

Male 10- to 12-week-old BALB/c mice were obtained from Nihon CLEA (Tokyo, Japan). Mice were maintained under standard conditions in accordance with our institutional guidelines. All animal experiments were approved by the Institutional Animal Care and Use Committee of Niigata University (SA00002). *S. pneumoniae* D39 (NCTC 7466) was grown in tryptic soy broth (Becton Dickinson, Franklin Lakes, NJ, USA). For *in vitro* cytokine assays, *S. pneumoniae* D39 was inactivated by heating at 60°C for 1 h.

### Cell Line

The monocytic cell line THP-1 was maintained in 25 mM HEPES-buffered RPMI 1640, supplemented with 10% fetal bovine serum, 100 U/mL penicillin, and 100 µg/mL streptomycin (Wako Pure Chemical Industries, Osaka, Japan) at 37°C in 95% air and 5% CO_2_. For the experiments, the cells were incubated in a 24-well culture plate at a concentration of 2 × 10^5^ cells/mL in medium supplemented with 200 nM phorbol 12-myristate 13-acetate to induce differentiation into macrophage-like cells, hereafter referred to as macrophages. After 48 h incubation, the cells were washed with RPMI 1640 and cultured further in RPMI 1640 for 12 h. Then, cells were stimulated with heat-killed *S. pneumoniae* (HK-Spn) or *Escherichia coli* lipopolysaccharide (LPS) (100 ng/mL; Sigma-Aldrich, St. Louis, MO, USA) in the presence or absence of human neutrophil elastase (hNE; Innovative Research, Novi, MI, USA) and the NE inhibitor sivelestat (100 µg/mL; ONO Pharmaceutical Co., Osaka, Japan) for 3 h under serum-free conditions.

### Quantitative Real-Time PCR

Gene transcription in THP-1-derived macrophages was quantified using quantitative real-time PCR. Briefly, RNA was extracted from cell lysates using TRI Reagent (Molecular Research Center, Inc., Cincinnati, OH, USA) and quantified by spectrometry at 260 and 280 nm. The RNA was reverse transcribed using SuperScript VILO Master Mix (Thermo Fisher Scientific, Waltham, MA, USA), and quantitative real-time PCR with cDNA was performed with the StepOnePlus real-time PCR system (Thermo Fisher Scientific) according to manufacturer protocol. TaqMan probes, sense primers, and antisense primers for expression of *GAPDH, TNF, IL6*, and *IL8* were purchased from Thermo Fisher Scientific.

### Immunofluorescence Analysis

Human neutrophil elastase-treated macrophages stimulated with HK-Spn or LPS were fixed and permeabilized using a cell fixation and permeabilization kit (Thermo Fisher Scientific) according to manufacturer instructions, followed by incubation of the cells in a blocking solution (Thermo Fisher Scientific) for 30 min. Samples were stained with rabbit anti-NF-κB p65 antibody (Santa Cruz Biotechnology, Dallas, TX, USA), anti-toll-like receptor (TLR) 2 antibody (clone TL2.1; Thermo Fisher Scientific), or anti-TLR4 antibody (clone HTA125; Thermo Fisher Scientific) in blocking solution. After overnight incubation at 4°C, secondary AlexaFluor 594-conjugated goat anti-rabbit IgG antibody or AlexaFluor 488-conjugated goat anti-mouse IgG antibody (Thermo Fisher Scientific) in blocking buffer was added, followed by a 2-h incubation in the dark. Then, samples were observed using a confocal laser-scanning microscope (Carl Zeiss, Jena, Germany). In addition, samples were observed with fluorescence microscopy using Hybrid cell-count software (Keyence, Osaka, Japan) to calculate fluorescence intensity per cell.

### Western Blot Analysis

Whole cell lysates were prepared in 100 µL of lysis reagent (Thermo Fisher Scientific). For recombinant proteins cleavage assay, 100 ng of recombinant human (rh) TLR2 (R&D Systems, Minneapolis, MN, USA), rhTLR4 (R&D Systems), rh-myeloid differentiation factor 2 (MD2) (Abcam, Cambridge, UK), rh-tumor necrosis factor (TNF) (R&D Systems), rhIL-6 (PeproTech, Rocky Hill, NJ, USA), or rhIL-8 (R&D Systems) was treated with various concentrations of hNE (125–500 mU/mL) in the presence or absence of the NE inhibitor sivelestat (100 µg/mL) at 37°C for 3 h and mixed with 2% SDS-sample buffer. All samples were separated by SDS-PAGE and transferred to polyvinylidene difluoride membranes (Merck Millipore, Billerica, MA, USA) followed by incubation with blocking reagent (Nacalai Tesque, Kyoto, Japan). The membrane was probed with an anti-TLR2 antibody (Rockland Immunochemicals, Limerick, PA, USA), anti-TLR4 antibody (Novus Biologicals, Littleton, CO, USA), anti-MD2 antibody (Abcam), anti-NF-κB antibody (Santa Cruz Biotechnology), anti-GAPDH antibody (Abcam), anti-TNF antibody (Cell Signaling Technology, Beverly, MA, USA), anti-IL-6-antibody (Abcam), or anti-IL-8 antibody (R&D Systems) and then incubated with a HRP-conjugated secondary antibody (Cell Signaling Technology) in Tris-buffered saline containing 0.05% tween 20. The membrane was treated with HRP substrates (GE Healthcare, Chicago, IL, USA) and analyzed using a chemiluminescence detector (Fujifilm, Tokyo, Japan).

### Cytokine Assay

The levels of TNF, IL-6, and IL-8 in the cell culture supernatants were determined by using enzyme-linked immunosorbent assay (ELISA) kits (BioLegend, San Diego, CA, USA).

### Bead-Based Multiplex Assay for Cytokines

Comprehensive hNE-induced cytokine cleavage assays were performed using the MILLIPLEX MAP human cytokine/chemokine magnetic bead panel (Merck Millipore) by determining 18 cytokine concentrations (G-CSF, GM-CSF, IFNα2, IFN-γ, IL-10, IL12p40, IL-12p70, IL-17, IL-1β, IL-2, IL-3, IL-4, IL-6, IL-8, MCP-1, MIP1α, MIP1β, and TNF). Human cytokine/chemokine cocktail (Merck Millipore) was treated with various concentrations of hNE (125–500 mU/mL) in the presence or absence of the NE inhibitor sivelestat (100 µg/mL) at 37°C for 3 h followed by addition of sivelestat (100 µg/mL; final concentration) to inactivate hNE. These samples were assayed according to the manufacturer’s instructions. Briefly, standards, controls, and samples were loaded into 96-well plates, followed by addition of antibody-immobilized beads. Plates were incubated for 2 h on a plate shaker and then washed twice with wash buffer. Detection antibodies were then added to the wells and incubated for 1 h. Finally, streptavidin-phycoerythrin was added to the wells and incubated for 30 min. Plates were gently washed twice with wash buffer before resuspending the beads in sheath fluid followed by analysis on a Luminex 200 (Merck Millipore). Using xPONENT software version 3.1 (Luminex Corporation, Austin, TX, USA), a standard curve was generated using a 5-parameter logistic curve for each mediator ranging from 10,000 to 3.2 pg/mL, and then cytokine concentrations were calculated.

### Intratracheal Infection of Pneumococcus *In Vivo*

Following mouse anesthesia with isoflurane using an inhalational anesthesia system (Natsume Seisakusho, Tokyo, Japan), the trachea was aseptically exposed and an *S. pneumoniae* inoculum [2 × 10^8^ colony forming units (CFUs) in 50 µL phosphate buffered saline (PBS)] was administered *via* a 26-gauge needle (20). Unchallenged naïve mice were administered PBS only. NE inhibitor (40 mg/kg) or PBS was administered intraperitoneally to the infected mice every 6 h. Groups of animals were sacrificed at 18 h postinfection. To obtain BALF, 1.0 mL PBS was instilled into mouse lungs and then slowly aspirated. BALF samples were then plated onto blood-agar plates and cultured aerobically for enumerating recovered CFU. To determine NE activity, cytokine levels, and recruitment of inflammatory cells, BALF was centrifuged at 500 *g*, and the supernatant was used for subsequent NE activity assay and cytokine analysis. The cell pellet was analyzed for total leukocyte count using Turk’s solution (Nacalai Tesque). NE activity in BALF was determined by a method using the NE-specific substrate *N*-methoxysuccinyl-Ala-Ala-Pro-Val *p*-nitroanilide (Merck Millipore) as described elsewhere (14). Briefly, samples were incubated in 0.1 M Tris–HCl buffer (PH 8.0) containing 0.5 M NaCl and 1 mM substrate at 37°C for 24 h, and then absorbance at 405 nm was measured. The levels of TNF and IL-6 in BALF and serum were determined by using ELISA kits (BioLegend). To determine the concentration of pneumococcal DNA, DNA was isolated from 50 µL of serum from the infected mice using QIAamp Spin Columns (QIAGEN, Hilden, Germany). Then, absolute quantification by real-time PCR was performed using the StepOnePlus real-time PCR system *via* the SYBR Green detection protocol. The primers used for real-time PCR were based on published sequence (21). The forward primer oligonucleotide sequence was 5′-AGCGATAGCTTTCTCCAAGTGG-3′ and the reverse primer sequence was 5′-CTTAGCCAACAAATCGTTTACCG-3′.

To determine the effect of the NE inhibitor on the mouse pneumococcal pneumonia model, mice were intratracheally infected with *S. pneumoniae* (5 × 10^8^ CFU in 50 µL PBS), and then the NE inhibitor (40 mg/kg) or PBS was administered intraperitoneally to these mice every 6 h. Survival was monitored following the treatments.

### Statistical Analysis

Data were analyzed statistically by analysis of variance with Tukey’s multiple-comparison test. Where appropriate (comparison of two groups only), unpaired *t* tests were conducted. The Kaplan–Meier survival curve was analyzed using the log-rank test equivalent to the Mantel–Haenszel test. All statistical analyses were performed using Graph Pad Prism Software version 6.05 (GraphPad Software, Inc., La Jolla, CA, USA). Values of *P* < 0.05 were considered significant.

## Results

### NE Downregulates Cytokine Gene Transcription in Macrophages by Inhibiting NF-κB Nuclear Translocation

**Figure 1:**
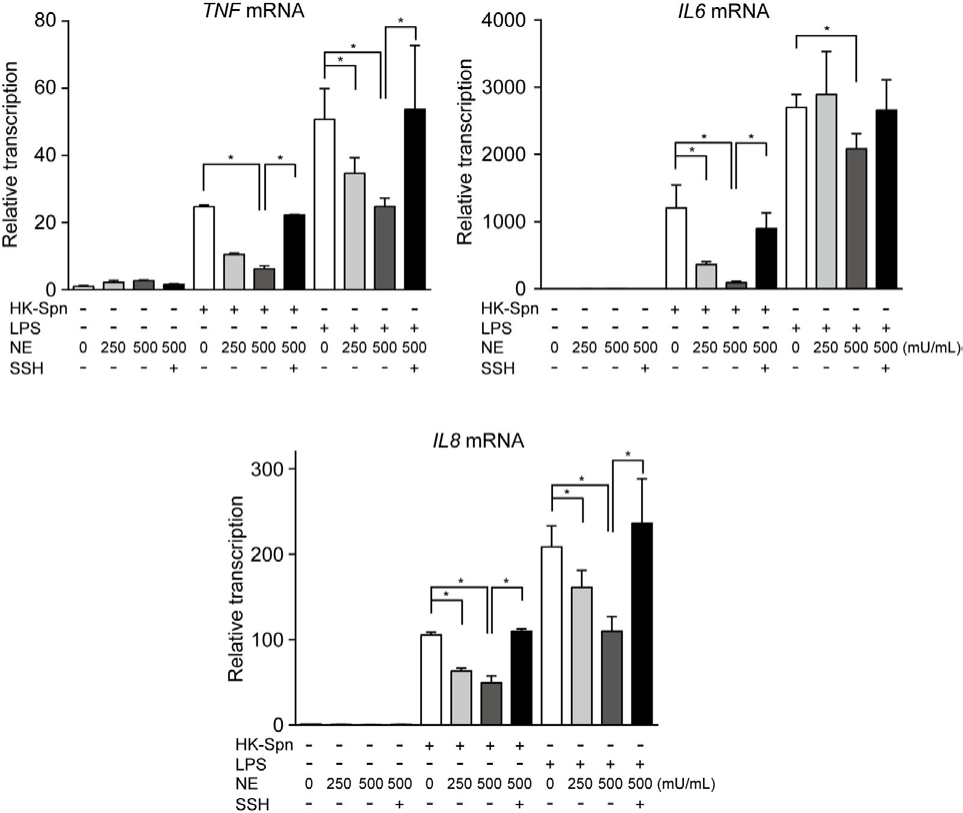
Neutrophil elastase (NE) downregulates cytokine gene transcription in macrophages stimulated with TLR agonists. THP-1-derived macrophages were stimulated with HK-Spn D39 or *Escherichia coli* LPS (100 ng/mL) in the presence or absence of hNE (250–500 mU/mL) and/or SSH (100 µg/mL) for 4 h under serum-free conditions. Real-time PCR was performed to quantify *TNF, IL6*, and *IL8* mRNA in the macrophages exposed to these stimuli. The relative quantity of these cytokine mRNAs was normalized to the relative quantity of *GAPDH* mRNA. Data represent the mean ± SD of quadruplicate experiments and were evaluated using one-way analysis of variance with Tukey’s multiple-comparisons test. *Significantly different within the same activation status at *P* < 0.05. HK-Spn, heat-killed *S. pneumoniae*; LPS, lipopolysaccharide; hNE, human neutrophil elastase; SSH, sivelestat sodium hydrate; TLR, toll-like receptor; TNF, tumor necrosis factor.

**Figure 2:**
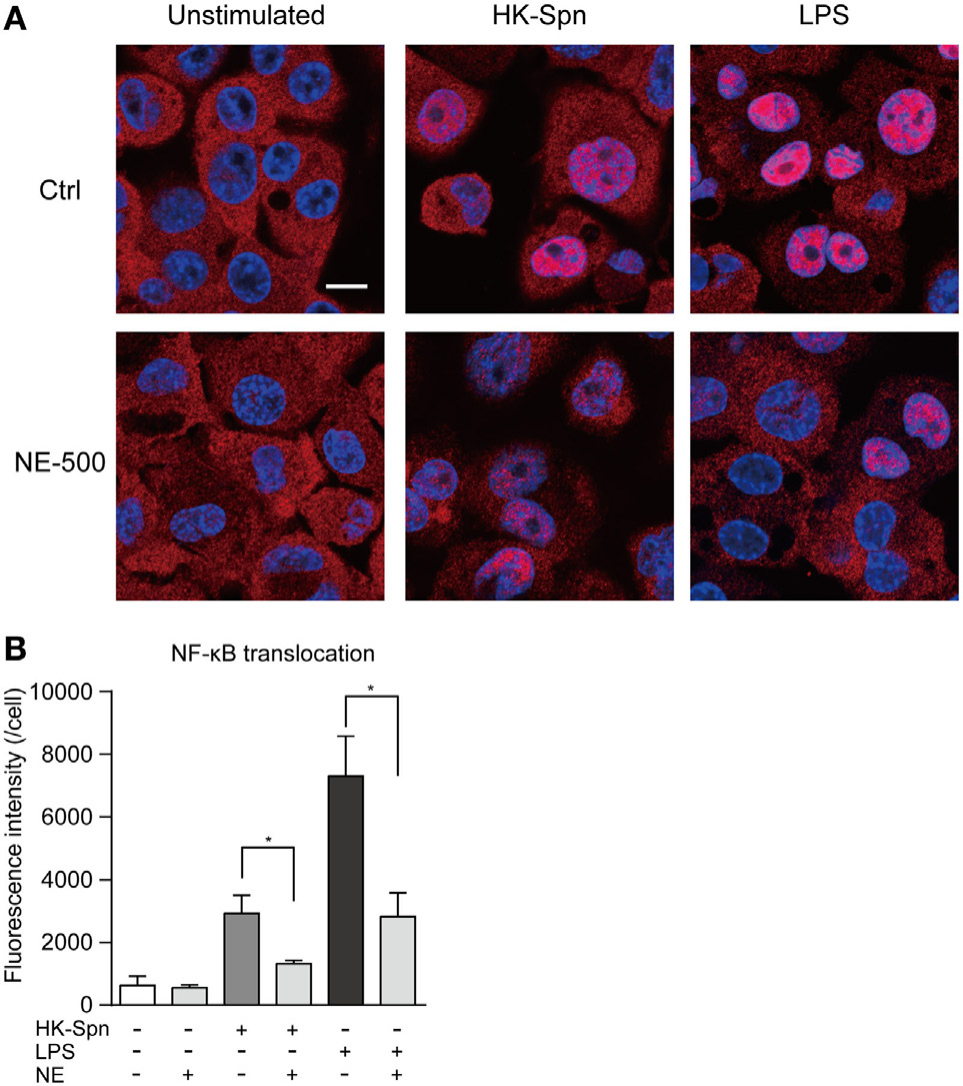
Neutrophil elastase (NE) inhibits NF-κB translocation to the nucleus in macrophages. THP-1-derived macrophages were exposed to hNE (500 mU/mL) in the presence or absence of HK-Spn or LPS (100 ng/mL) for 30 min. **(A)** Representative fluorescence microscopy images of macrophages stained for DNA (DAPI; blue) and NF-κB p65 (red). Scale bar: 10 µm. **(B)** The fluorescence intensity of NF-κB p65 in the nucleus per cell was calculated. Data represent the mean ± SD of quadruplicate experiments and were evaluated using one-way analysis of variance with Tukey’s multiple-comparisons test. *Significantly different within the same activation status at *P* < 0.05. HK-Spn, heat-killed *S. pneumoniae*; LPS, lipopolysaccharide; hNE, human neutrophil elastase.

We first investigated whether hNE affects cytokine gene transcription in macrophages. Accordingly, macrophages were stimulated with HK-Spn and LPS, which are recognized by TLR2 (22) and TLR4 (23), respectively, in the presence or absence of hNE. Strikingly, hNE significantly and dose-dependently reduced HK-Spn- and LPS-induced *TNF, IL6*, and *IL8* gene transcription in the macrophages, with the exception of *TNF* and *IL6* in the cells treated with 250 mU/mL hNE (Figure 1). Furthermore, downregulation of these cytokines in hNE-treated macrophages was counteracted by the administration of sivelestat. hNE did not show cytotoxicity toward macrophages up to 500 mU/mL (Figure S1 in Supplementary Material). As hNE downregulated the transcription of various cytokine genes, we hypothesized that hNE inhibits TLR signaling in the macrophages. Therefore, we next examined whether hNE could inhibit NF-κB nuclear translocation in the macrophages stimulated with HK-Spn or LPS. Although HK-Spn and LPS-induced NF-κB activation and subsequent nuclear translocation, 500 mU/mL hNE significantly inhibited the latter (Figures 2A,B), suggesting that hNE-induced inhibition of TLR signaling can downregulate cytokine gene transcription.

### NE Cleaves TLRs and MD2 on Macrophages

**Figure 3:**
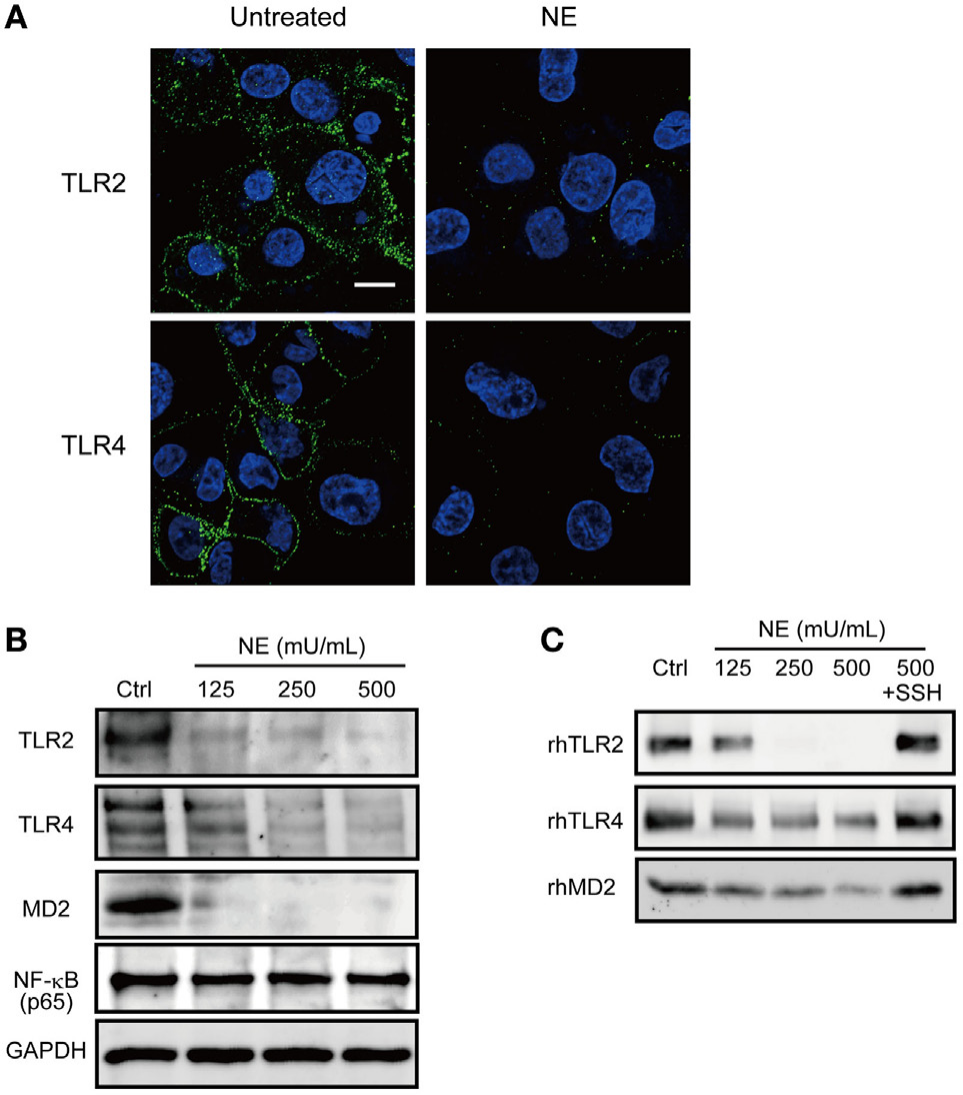
Neutrophil elastase (NE) cleaves TLRs and MD2 on macrophages. **(A,B)** THP-1-derived macrophages were cultured in serum free RPMI 1640 and exposed to hNE (125–500 mU/mL) for 4 h. **(A)** Representative fluorescence microscopy images of untreated and hNE-treated (500 mU/mL) macrophages stained for DNA (DAPI; blue) and TLRs (green). **(B)** TLR2, TLR4, and TLR-related molecules were determined by western blot analysis. **(C)** Recombinant human (rh) TLR2, rhTLR4, and rhMD2 were exposed to hNE (125–500 mU/mL) in the presence or absence of SSH (100 µg/mL) for 3 h and determined by western blot analysis. MD2, myeloid differentiation factor 2; hNE, human neutrophil elastase; SSH, sivelestat sodium hydrate; TLR, toll-like receptor.

It has been reported that NE mediates intracellular signaling through proteinase-activated receptor (PAR)-1 or PAR-2 (24, 25). These findings prompted us to further examine whether NE-dependent activation of these receptors is involved in the downregulation of cytokine gene transcription. However, neither a PAR-1 antagonist (SCH79797) nor PAR-2 antagonist (FSLLRY-NE2) could counteract the downregulation of *TNF* gene transcription in the hNE-treated macrophages (Figure S2 in Supplementary Material), indicating that the effect of hNE is not mediated by PARs. Given that NE can cleave some cell-surface receptors, such as urokinase receptor and C5a receptor (9, 26), we investigated whether hNE cleaves TLRs. Indeed, hNE decreased the protein expression of TLR2, TLR4, and MD2 in hNE-treated macrophages (Figures 3A,B). Moreover, we observed that rhTLR2, rhTLR4, and rhMD2 were cleaved by hNE after 3 h of coincubation (Figure 3C). Taken together, our findings indicate that hNE inhibits TLR signaling by cleaving TLRs and MD2.

### NE Also Cleaves Various Proinflammatory Cytokines *In Vitro*

**Figure 4:**
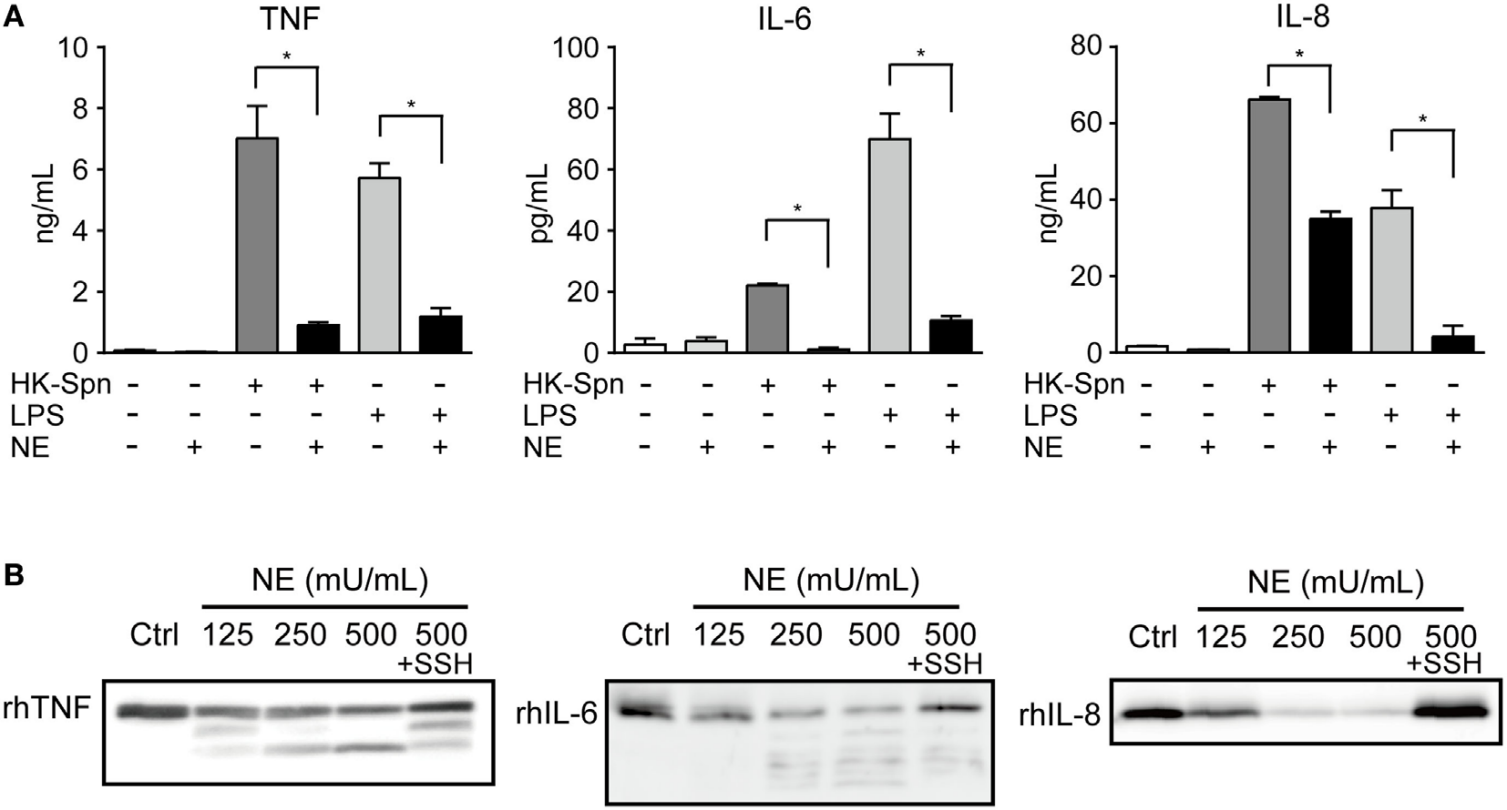
Neutrophil elastase (NE) degrades TNF, IL-6, and IL-8 **(A)**. THP-1-derived macrophages were exposed to hNE (125–500 mU/mL) in the presence or absence of HK-Spn or LPS (100 ng/mL) for 4 h. TNF, IL-6, and IL-8 concentrations in the culture supernatants were determined by ELISA. Data represent the mean ± SD of quadruplicate experiments and were evaluated using one-way analysis of variance with Tukey’s multiple-comparisons test. *Significantly different within the same activation status at *P* < 0.05. **(B)** rhTNF, rhIL-6, and rhIL-8 were exposed to hNE (125–500 mU/mL) in the presence or absence of SSH (100 µg/mL) for 3 h and determined by western blot analysis. ELISA, enzyme-linked immunosorbent assay; HK-Spn, heat-killed *S. pneumoniae*; LPS, lipopolysaccharide; hNE, human neutrophil elastase; rh, recombinant human; SSH, sivelestat sodium hydrate; IL, interleukin; TNF, tumor necrosis factor.

To confirm hNE-induced inhibition of TLR signaling, we measured *in vitro* cytokine levels (TNF, IL-6, and IL-8) in culture supernatants of HK-Spn- or LPS-stimulated macrophages in the presence of absence of hNE. Consistent with the repression of cytokine gene transcription, treatment of the macrophages with hNE resulted in marked reduction of these cytokine levels in the supernatant (Figure 4A). Among these, IL-6 levels were almost completely abolished in the supernatant of hNE-treated macrophages stimulated with HK-Spn. We thus speculated that hNE cleaves not only TLRs but also proinflammatory cytokines. To test this, rhTNF, rhIL-6, and rhIL-8 were treated with varying concentrations of hNE in the presence or absence of sivelestat. Figure 4B shows that hNE cleaved these cytokines, whereas this effect was counteracted by sivelestat. Additionally, rhTNF and rhIL-6 that had been treated by hNE exhibited lower-molecular-mass fragments. We next evaluated the effect of hNE on the cytokine levels in the supernatant from mouse peritoneal macrophages infected with *S. pneumoniae*. Figure S3 in Supplementary Material shows that hNE treatment almost completely obliterated mouse IL-6 in the supernatant, which is consistent with the results shown in Figure 4A, whereas hNE treatment did not decrease mouse TNF levels. These findings suggest the possibility that mouse proteins show different sensitivity to hNE-induced proteolysis as compared with human proteins.

**Figure 5:**
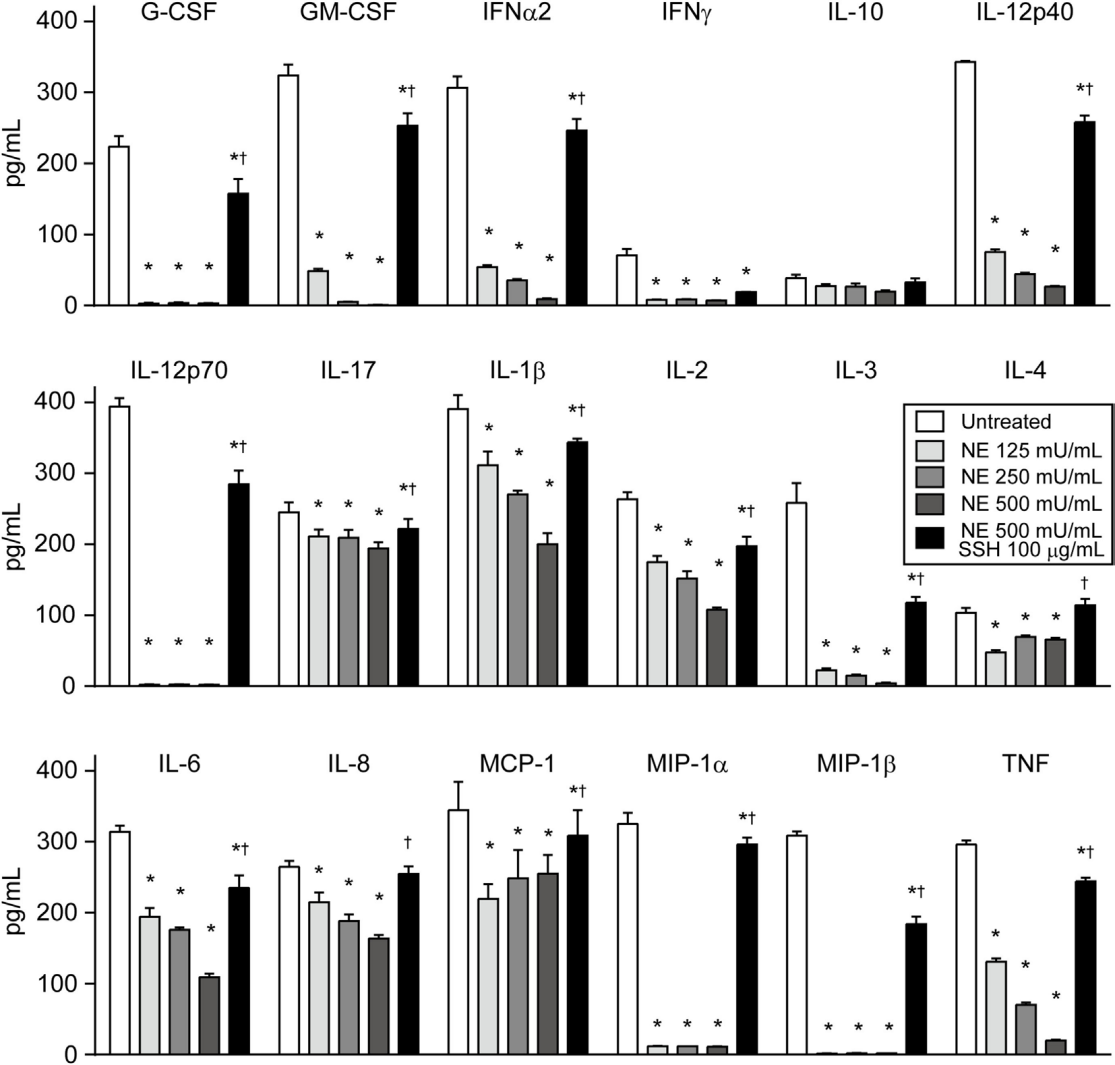
Multiplex assay reveals that neutrophil elastase (NE) degrades various cytokines. Human cytokine/chemokine cocktail was exposed to hNE (125–500 mU/mL) in the presence or absence of SSH (100 µg/mL) for 3 h. Then, cytokines were detected using a multiplex assay. Data represent the mean ± SD of quadruplicate experiments and were evaluated using two-way ANOVA. *Significantly different from the hNE-untreated control at *P* < 0.05. ^†^Significantly different from the hNE-treated (500 mU/mL) group in the absence of SSH at *P* < 0.05. hNE, human neutrophil elastase; SSH, sivelestat sodium hydrate.

Although the role of cytokines in pneumonia is not fully understood, various cytokines may be involved in innate and acquired pulmonary defense (1). Therefore, we comprehensively investigated the cleavage activity of hNE on 18 cytokines. Of these, 17 cytokine levels were significantly decreased by hNE treatment but showed different susceptibility to hNE (Figure 5). For example, IL-17 level was decreased by 20% in 500 mU/mL hNE, whereas G-CSF, IL-12p70, MIP-1α, and MIP-1β levels were almost abolished by 125 mU/mL hNE. Conversely, IL-10 was not significantly affected by hNE. These findings suggest that hNE cleaves a variety of cytokines and may subvert host immune responses during lung infections.

In contrast to our findings, it has been reported that NE treatment itself induces *IL8* gene transcription and protein release in alveolar epithelial cells and bronchial epithelial cells in the culture medium containing 10% fetal bovine serum (27, 28); however, 125 mU/mL hNE treatment did not result in an increase in IL-8 protein levels in the supernatant of A549 alveolar epithelial cells under serum-free conditions (Figure S4A in Supplementary Material). Higher concentrations (>100 mU/mL) of hNE induced the detachment of A549 cells as described previously (19). The catalytic function of NE is blocked by serum α1-antitrypsin (29); therefore, we treated A549 cells with hNE under serum-containing conditions. Figure S4B in Supplementary Material shows that treatment with excessive concentrations (2,000–5,000 mU/mL) of hNE significantly increased IL-8 protein levels under serum-containing conditions, whereas treatment with 500–1,000 mU/mL hNE did not.

### Administration of an NE Inhibitor Increased BALF Cytokine Level and Decreased Bacterial Load *In Vivo*

**Figure 6:**
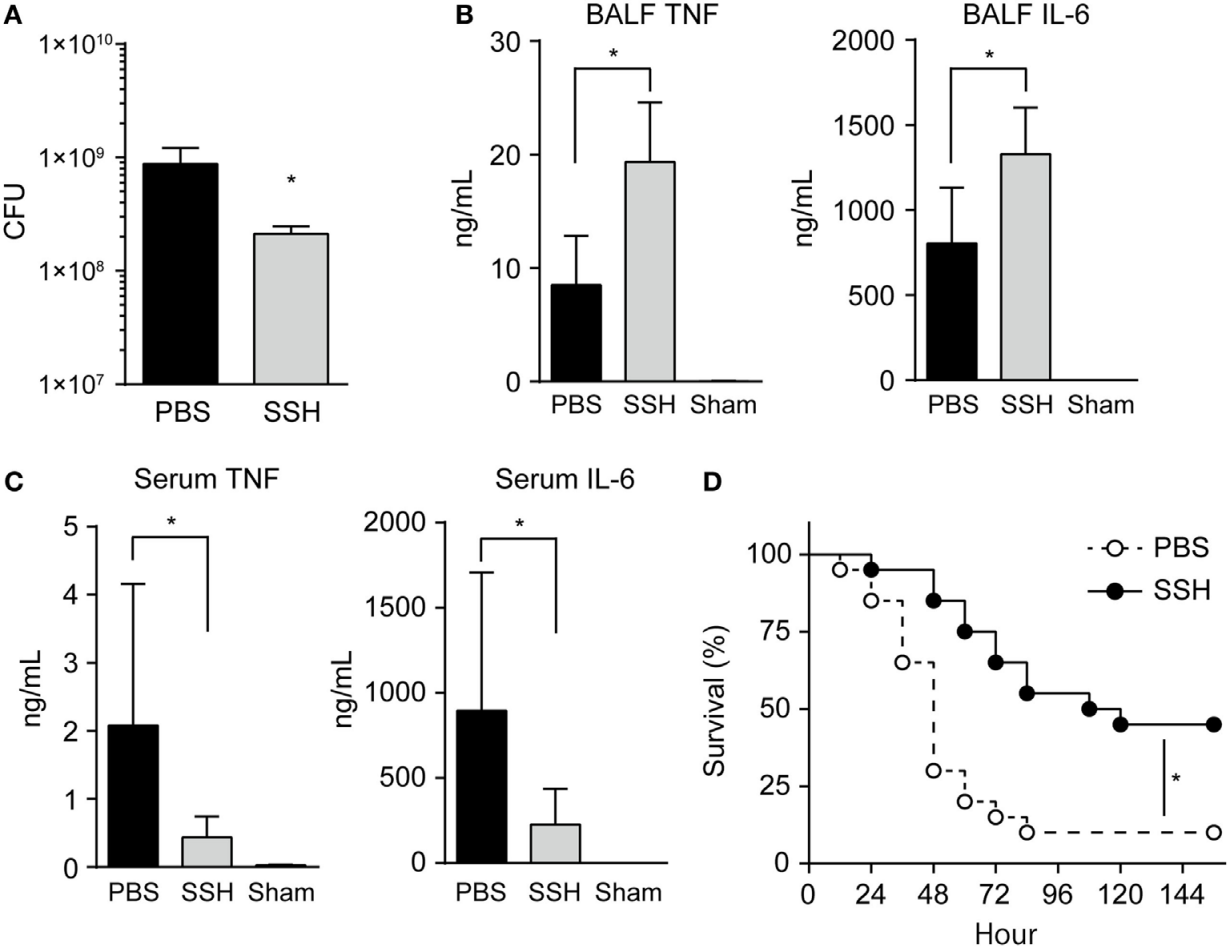
Administration of NE inhibitor increases cytokine level and decreases bacterial load in BALF. BALB/c mice (seven mice each) were intratracheally infected with *Streptococcus pneumoniae* D39 (2 × 10^8^ CFU in 50 µL PBS). Unchallenged naive mice (Sham group) were administered PBS only. NE inhibitor (SSH group; 50 mg/kg) or PBS (PBS group) was administrated intraperitoneally to the infected mice every 6 h. **(A–C)** Groups of animals were sacrificed at 18 h postinfection. **(A)** BALF samples were plated onto blood-agar plates and cultured aerobically for enumerating recovered CFU. Data represent the mean ± SD and were evaluated using unpaired *t* tests. *Significantly different from the infected control group at *P* < 0.05. **(B)** The levels of TNF and IL-6 in BALF were determined by using ELISA kits. **(C)** Serum TNF and IL-6 levels were determined. **(B,C)** Data represent the mean ± SD and were evaluated using one-way analysis of variance with Tukey’s multiple-comparisons test. *Significantly different from the infected control group at *P* < 0.05. **(D)** Survival of BALB/c (twenty mice each) mice was monitored following the intratracheal infection with *S. pneumoniae* (5 × 10^8^ CFU) in the presence or absence of intraperitoneal administration of an NE inhibitor. Statistical analysis was performed with log-rank test. *Significantly different from the SSH-untreated control at *P* < 0.001. BALF, bronchoalveolar lavage fluid; CFUs, colony forming units; ELISA, enzyme-linked immunosorbent assay; NE, neutrophil elastase; PBS, phosphate buffered saline; SSH, sivelestat sodium hydrate; IL, interleukin; TNF, tumor necrosis factor.

To confirm whether NE alters cytokine homeostasis and induces immune subversion *in vivo*, we investigated the effect of NE in mice after intratracheal infection with pneumococcus, in the presence or absence of sivelestat. First, we confirmed that sivelestat does not possess direct antimicrobial activity against *S. pneumoniae* (Figure S5 in Supplementary Material). Although pulmonary infection causes the infiltration of neutrophils and serum proteins, including α1-antitrypsin, into lungs (30), NE activity was significantly elevated in BALF from sivelestat-untreated mouse compared with uninfected mouse (sham) at 18 h postinfection (average: 431.6 vs. 2.6 mU/mL; Figure S6A in Supplementary Material). This data suggests that increased IL-8 protein levels in the supernatant due to ≥2,000 mU/mL hNE-treated cells under the serum-containing condition *in vitro* likely does not reflect the *in vivo* status. Figure S6A in Supplementary Material also shows that BALF from sivelestat-treated mice contained significantly lower NE activity compared to that of untreated control mice (>65% reduction). In addition, sivelestat treatment significantly decreased pneumococcal CFU in BALF (>65% reduction; Figure 6A). By contrast, IL-6 and TNF levels were significantly higher in sivelestat-treated BALF (Figure 6B). These data suggest that NE-dependent cleavage of these cytokines was inhibited following treatment with sivelestat, resulting in the apparent increase in the BALF cytokine level. Sivelestat treatment did not affect the number of leukocytes in BALF (Figure S6B in Supplementary Material). Although hNE reduced cytokine gene transcription in the macrophages *in vitro*, the *Il6* and *Tnf* gene transcription levels in whole lung samples appeared similar between untreated and sivelestat-treated mice (Figure S6C in Supplementary Material); this observation could be attributed to the higher bacterial loads in untreated control mice. These data indicate that decreased proinflammatory cytokine levels in sivelestat-untreated mice were mainly attributed to the NE-dependent cleavage of cytokines in this mouse model.

A recent human study demonstrated that serum IL-6 and TNF levels are associated with early mortality of patients with pneumonia (31). Therefore, we next investigated whether higher IL-6 and TNF levels in BALF from sivelestat-treated mice are correlated with these cytokine levels in serum samples. However, IL-6 and TNF levels were significantly lower in the serum from sivelestat-treated mice (Figure 6C). The significantly higher serum cytokine levels in untreated control mice, which contradicts the NE-induced lower cytokine levels in BALF, is partly explained by the α1-antitrypsin-mediated blockade of NE activity in serum. Additionally, the untreated control mice were found to be bacteremic for *S. pneumoniae* (pneumococcal DNA detection in four out of seven blood samples in this group), whereas lower prevalence of bacteremia (one out of seven blood samples) could be detected in the sivelestat-treated group. Serum pneumococcal DNA concentration correlated directly with serum IL-6 (*r* = 0.82, *P* < 0.001) and TNF (*r* = 0.88, *P* < 0.001) levels after inoculation with *S. pneumoniae* (Figure S6D in Supplementary Material). These data suggest that pneumococcal bacteremia induced increases in serum cytokine levels in untreated control mice. Figure 6D illustrates the cumulative survival of mice after intratracheal infection. The mortality of sivelestat-treated mice inoculated with *S. pneumoniae* was significantly lower than that of untreated control mice (*P* = 0.001); in particular, 90% (18/20) of the untreated mice died by 4 days after infection, whereas 55% (11/20) of sivelestat-treated mice dead within 5 days of infection. These *in vivo* findings demonstrate that the subversion of host immune responses by NE causes bacterial invasion of the bloodstream followed by death.

## Discussion

**Figure 7:**
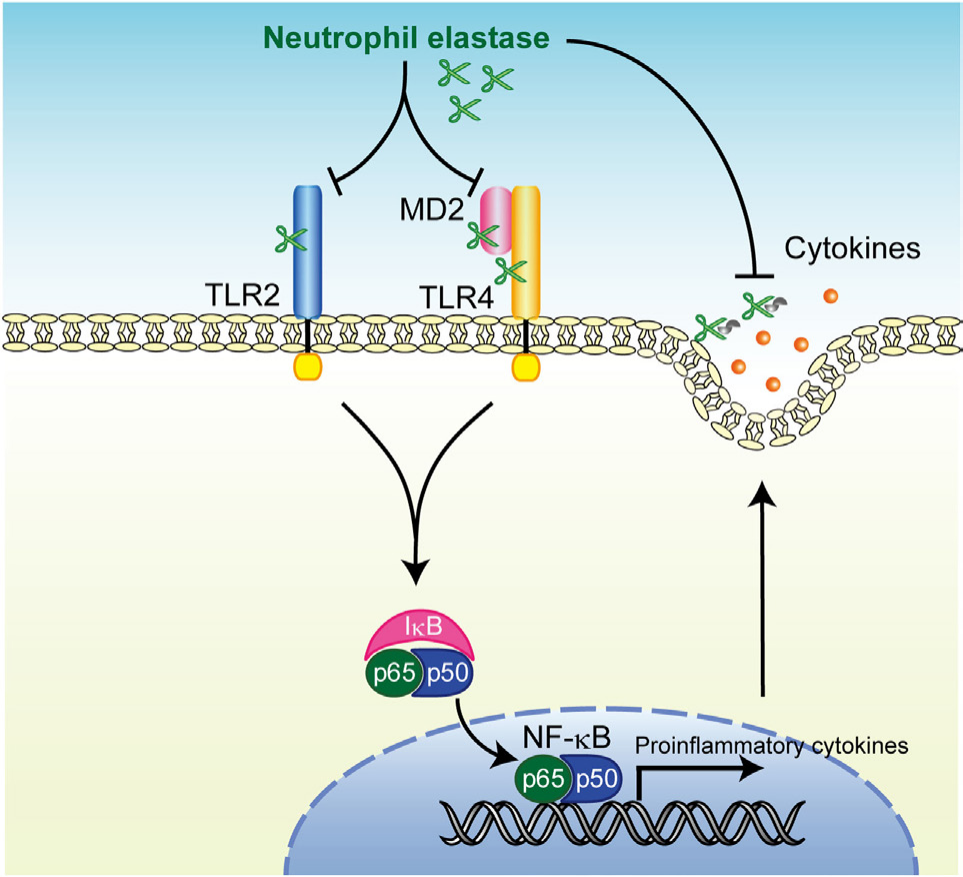
Summary of neutrophil elastase-induced suppression of toll-like receptor (TLR) signaling. Human neutrophil elastase (hNE) degrades TLRs and myeloid differentiation factor 2 (MD2) followed by inhibition of downstream signaling pathways including NF-κB activation, which subsequently downregulates cytokine gene transcription. hNE also degrades secreted cytokines and eventually impairs the host innate immune response.

Innate immune defenses are primarily responsible for the clearance of foreign particles deposited on the surface of airways and elimination of bacterial pathogens from the alveolus (32). The development of pneumonia indicates a defect in host defense, exposure to an overwhelming inoculum of virulent microorganism, or a combination of these factors (33). Here, we addressed the intriguing possibility that at least some of the innate immune receptors, such as TLR2 and TLR4, were cleaved by NE, which was originally identified as a powerful host defense component, leading to the inhibition of downstream signaling pathways followed by downregulation of cytokine gene transcription (Figure 7). Furthermore, NE also cleaves various cytokines secreted from macrophages in response to TLR agonists and leads to the impairment of host innate immune defenses and death. The pneumococcal intracellular toxin, pneumolysin, constitutes such an NE-inducing factor through its ability to promote pore formation in the neutrophilic plasma membrane (19). Together, these observations indicate that pneumococcus exploits NE as an etiological agent during pneumonia.

Toll-like receptors are key molecules that recognize pathogen-associated molecular patterns and induce an inflammatory response (23). Among these, TLR2 and TLR4 are thought to be critical in bacterial infections and have been studied in pneumococcal pneumonia. Although TLR2, which recognizes various pathogen-associated molecular patterns of Gram-positive microbes, plays a limited role in the survival of a mouse pneumococcal pneumoniae model, the responsiveness of alveolar macrophages against *S. pneumoniae* depends on the presence of TLR2 (34). In addition to LPS of Gram-negative bacteria, pneumococcal pneumolysin is also considered to be a TLR4 ligand (35). Accordingly, TLR4-deficient mice showed impaired antibacterial defense against *S. pneumoniae* and reduced survival (36). In a human study, genetic variability in the *TLR4* gene was associated with an increased risk of developing invasive disease in patients infected with *S. pneumoniae* (37). These findings indicate that TLR2, TLR4, or a combination of these receptors play an essential role in pneumococcal pneumonia; thus, it is consistent that cleavage of these receptors by NE results in impaired immune responses and decreased survival for pneumococcal infections.

Several studies have demonstrated the importance of proinflammatory cytokines in host defense during pneumonia and other infectious diseases. TNF activates phagocytosis, oxidative burst, and bacterial killing (38). Our previous findings of NE-induced impairment of phagocytosis in macrophages (19) suggest that the NE-dependent cleavage of TNF and other cytokines observed in the present study may mediate the reduction of phagocytic activity, which leads to the increment of bacterial load in the lungs of NE inhibitor-untreated mice. Consistent with this, it has been reported that treatment with neutralizing anti-TNF monoclonal antibody resulted in an enhanced outgrowth of *S. pneumoniae* in the lungs and blood, along with significantly earlier death in mice with pneumococcal pneumonia as compared with control mice (39). Moreover, IL-6 and IFN-γ knockout mice showed impaired defense against pneumococcal pneumonia and demonstrated higher mortality (40, 41). Chemokines, such as KC, MCP-1, and MIP-1α, contribute to pulmonary neutrophil recruitment and macrophage infiltration (42). G-CSF- and GM-CSF-treated mice showed improved lung clearance of pneumococci, suggesting the protective role of these cytokines in pneumonia (43, 44). Although cytokine and chemokine networks in pneumococcal pneumonia are highly complex and not fully understood, NE-induced dysregulation of these networks may cause the disruption of pulmonary homeostasis, which leads to bacterial growth in the lungs thereby potentially causing death.

Neutrophil elastase has been recognized as a double-edged sword of innate immunity as it can act as both a host defensive and tissue destructive factor (13). Although NE plays an important role in intracellular pneumococcal killing by neutrophils (45), leakage of NE into the extracellular space promotes lung injury without killing the pneumococcal cells (19). In recent years, several experimental NE inhibitors, including sivelestat with low cellular permeability, have been tested in animal studies of pneumococcal pneumonia, with positive results (15). However, the mechanisms by which NE inhibitors improve survival rates in animal pneumonia models were not fully understood. One possible mechanism was that the NE inhibitor blocks NE-induced disruption of pulmonary epithelial cells and prevents bacterial invasion into bloodstream (14, 19). However, this alone cannot explain how an NE inhibitor enhanced clearance of pneumococci in the lung in this and previous studies (13). Our findings suggest that the NE inhibitor abolishes the effect of extracellular NE and subversion of the host innate immune response without impairing intracellular pneumococcal killing. Accordingly, we observed lower serum cytokine levels associated with decreased prevalence of bacteremia in sivelestat-treated mice as compared with untreated control mice.

In the present study, 40 mg/kg sivelestat were intraperitoneally administered to mice every 6 h, resulting in significantly decreased NE activity in BALF and markedly reduced mortality of mice inoculated with *S. pneumoniae* compared with untreated control mice. Yanagihara et al. demonstrated that administration of sivelestat (30 mg/mL per 12 h) resulted in moderately delayed mortality in pneumococcal pneumonia mouse model (14), whereas Mikumo et al. reported that administration of 150 mg/kg/day of sivelestat ameliorates pneumonitis and that there is significant improvement in the survival of mice administered naphthalene and gefinitib (46). Higher dose or increased frequency of administration could have effectively blocked NE activity in the present study. Further investigation is required to evaluate the optimal dose and frequency of administration of sivelestat.

Reportedly, NE-deficient mice in a model of *P. aeruginosa*-induced pneumonia showed that the absence of NE significantly increased bacterial CFU in BALF, which is caused by NE-mediated killing of Gram-negative bacteria by neutrophils in wild-type mice (47). Consistent with our findings, the same group also demonstrated the possibility of NE-dependent cleavage of TLR4 protein in wild-type mice (48). These data led us to predict the elevation of inflammatory cytokines in the BALF from the pneumonia model of NE-deficient mice. However, NE deficiency was associated with decreased levels of proinflammatory cytokines, such as TNF and IL-6, in BALF, which is inconsistent with our findings (48). This discrepancy may be caused by *P. aeruginosa*-induced proteolytic inactivation of these cytokines. *P. aeruginosa* possesses several proteases, including alkaline protease, elastase A, elastase B, and protease IV, that have been isolated and shown to be involved in pathogenesis. Additionally, *P. aeruginosa* produces two other proteases: modified elastase and *P. aeruginosa* small protease (49). Of these proteases, the cytokine degradation activities of alkaline protease and elastase B were reported (50). Although the *P. aeruginosa* strain 103, which was inoculated into NE-deficient mouse (47), is a low-protease-producing strain (51), it is possible that increased bacterial loads in the lungs of NE-deficient mice resulted in the elevation of local protease activity responsible for the degradation of host cytokines.

Although numerous studies have focused on the biological functions of hNE, little is known about how mouse NE acts *in vitro*. Although we demonstrated that hNE cleaved human TLRs and TNF, hNE did not significantly decrease mouse TNF levels *in vitro*. In an *in vivo* mouse intratracheal infection model, specific inhibition of NE by sivelestat significantly upregulated BALF TNF levels, suggesting that mouse NE cleaves mouse TNF. These results suggest that the peptide substrate specificities of human and mouse NE are different despite their conserved catalytic triad and close structural resemblance (52). In this regard, it has been reported that mouse and human NE may generate different peptide profiles from a common substrate (53). Therefore, there are limitations in the use of mouse models for studying NE pathogenesis for understanding human disease.

To our knowledge, this is the first study that has comprehensively examined the impact of NE on host innate immune receptors and cytokines. The NE inhibitor restored alveolar homeostasis and induced intrinsic innate immune responses during pneumococcal pneumonia. From a therapeutic viewpoint and in addition to antibiotic use, novel strategies for pneumonia treatment might include restraint of excessive neutrophil infiltration or NE-specific inhibition (13, 15, 54). Our findings further suggested that the evolutionary progression of pneumococcus has allowed the bacterium to exploit host molecules for developing severe pneumonia.

## Ethics Statement

Mice were maintained under standard conditions in accordance with our institutional guidelines. All animal experiments were approved by the Institutional Animal Care and Use Committee of Niigata University (SA00002).

## Author Contributions

HD and YT designed the study. HD, TM, KN, TH, HT, and WT performed all of the experiments. HD, MO, DY, MY, and SK analyzed the data. MY and SK prepared the materials. HD and YT wrote the paper. All authors discussed the results and approved the manuscript.

## Conflict of Interest Statement

The authors declare that the research was conducted in the absence of any commercial or financial relationships that could be construed as a potential conflict of interest.

## Abbreviations

BALF, bronchoalveolar lavage fluid; CFU, colony forming units; ELISA, enzyme-linked immunosorbent assay; HK-Spn, heat-killed *S. pneumoniae*; IL, interleukin; LPS, lipopolysaccharide; MD2, myeloid differentiation factor 2; hNE, human neutrophil elastase; PBS, phosphate buffered saline; rh, recombinant human; SSH, sivelestat sodium hydrate; TLR, toll-like receptor; TNF, tumor necrosis factor.
