# Supplementary material for: Neutrophil Elastase Subverts the Immune Response by Cleaving Toll-Like Receptors and Cytokines in Pneumococcal Pneumonia
Source: Front Immunol. 2018 Apr 25;9:732. doi: 10.3389/fimmu.2018.00732 (PMC5996908; doi:10.3389/fimmu.2018.00732)
Supplement: Supplementary file 1 [file Data_Sheet_1.PDF]

Supplementary Material

Neutrophil Elastase Subverts the Immune Response by Cleaving Toll-like Receptors and Cytokines in Pneumococcal Pneumonia

Hisanori Domon, Kosuke Nagai, Tomoki Maekawa, Masataka Oda, Daisuke Yonezawa, Wataru Takeda, Takumi Hiyoshi, Hikaru Tamura, Masaya Yamaguchi, Shigetada Kawabata, Yutaka Terao*

*** Correspondence:** Prof. Yutaka Terao: terao@dent.niigata-u.ac.jp

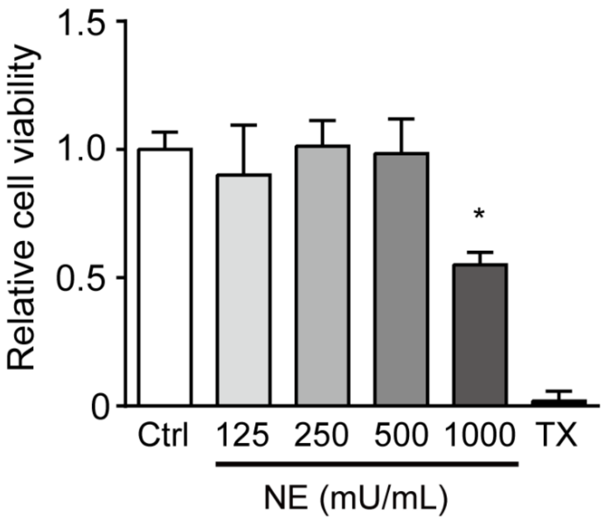

Figure S1. Effect of hNE on macrophage cell viability

THP-1-derived macrophages were exposed to various concentrations of hNE (125–1000 mU/mL) or 0.5% Triton X-100 for 6 h, followed by evaluation using an AlamarBlue cell viability assay (Thermo Fisher Scientific). Data represent the means ± SD of quadruplicate experiments and were evaluated using one-way analysis of variance with Dunnett's multiple-comparisons test. *Significantly different from the control group at $P < 0.05$. Ctrl, control; hNE, human neutrophil elastase; SD, standard deviation; TX, Triton X-100.

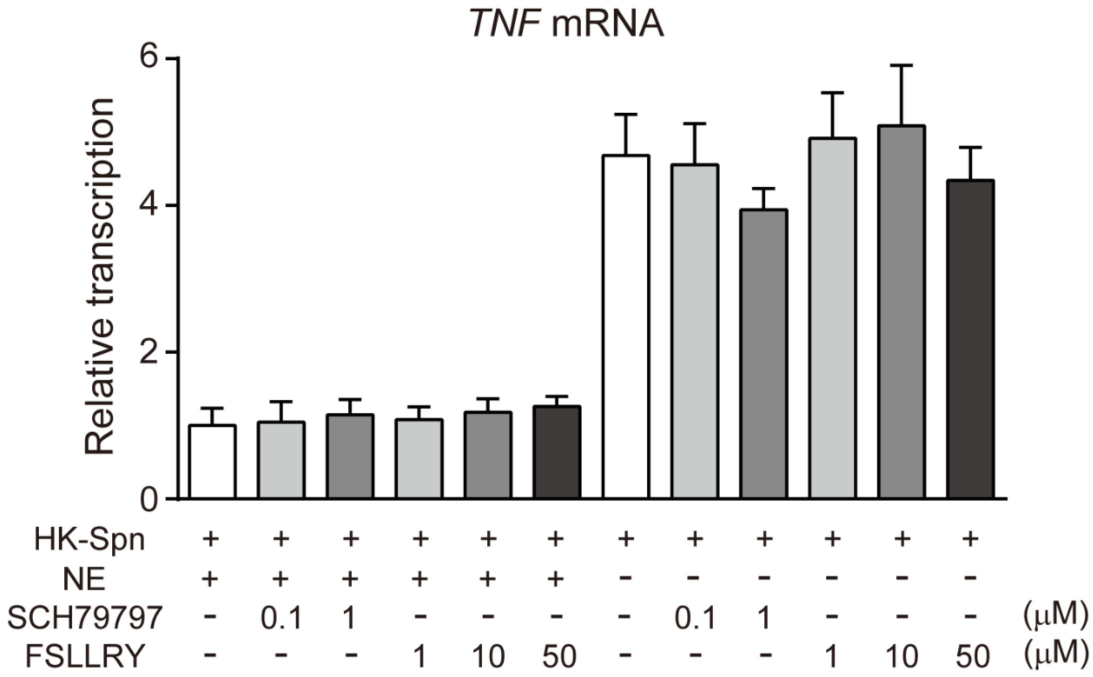

Figure S2. Proteinase-activated receptor is not involved in hNE-induced down-regulation of *TNF* gene transcription

THP-1-derived macrophages were pretreated with a PAR-1 antagonist (SCH79797; R&D Systems) or PAR-2 antagonist (FSLLRY-NE2; R&D Systems) for 1 h. The cells were then stimulated with HK-Spn in the presence or absence of 500 mU/mL hNE for 30 min. Real-time PCR was performed to quantify *TNF* mRNA. The relative quantity of *TNF* mRNA was normalized to the relative quantity of *GAPDH* mRNA. Data represent the means ± SD of quadruplicate experiments and were evaluated using one-way analysis of variance with Tukey's multiple-comparisons test. HK-Spn, heat-killed *S. pneumoniae*; hNE, human neutrophil elastase; SD, standard deviation.

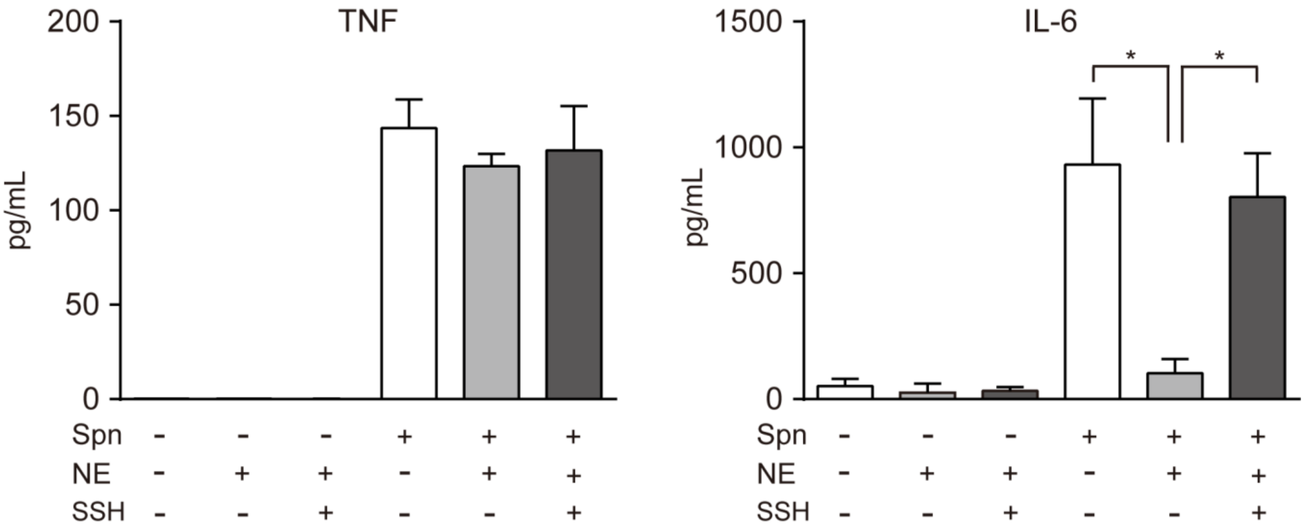

Figure S3. Effect of the human neutrophil elastase on TNF and IL-6 levels in the supernatant from mouse macrophages infected with *S. pneumoniae*.

Peritoneal macrophages (1×10^6 cells/500 µL) from BALB/c mice were infected with *S. pneumoniae* D39 (MOI = 10) in the presence or absence of 500 mU/mL hNE and/or 100 µg/mL SSH for 6 h under serum-free conditions. TNF and IL-6 concentrations in the culture supernatants were determined by ELISA kits (BioLegend, San Diego, CA, USA). Data represent the means ± SD of quadruplicate experiments and were evaluated using one-way analysis of variance with Tukey's multiple-comparisons test. *Significantly different between indicated groups at $P < 0.05$. ELISA, enzyme-linked immunosorbent assay; hNE, human neutrophil elastase; SD, standard deviation; Spn, *S. pneumoniae*; SSH, sivelestat sodium hydrate.

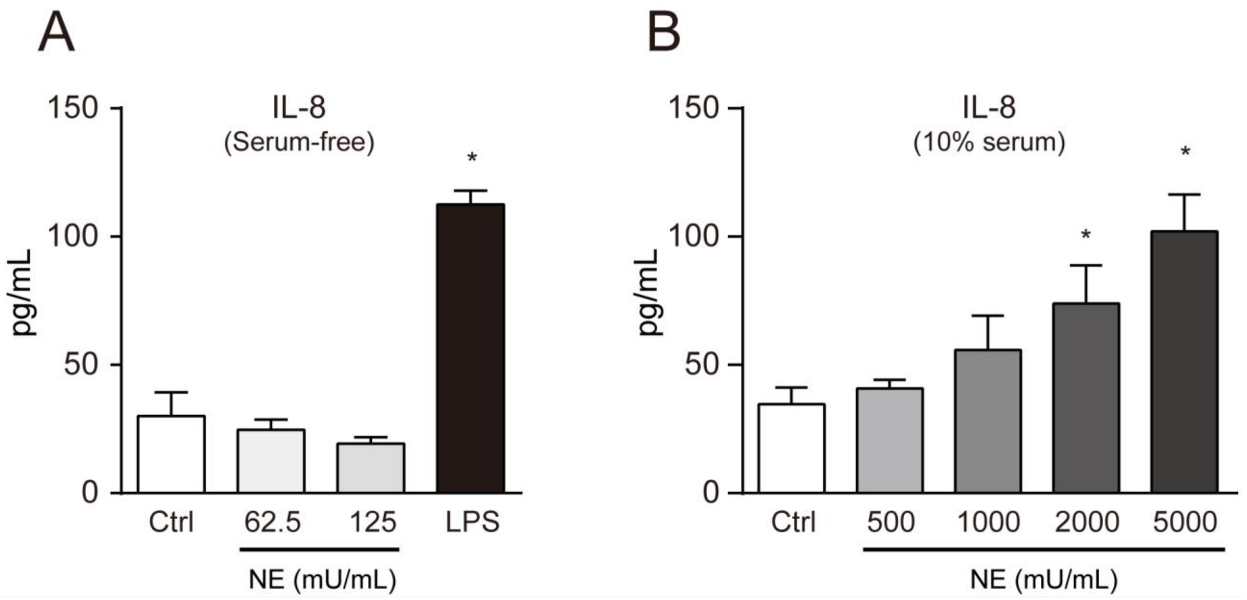

Figure S4. A549 cells induce IL-8 production in response to hNE in serum-containing medium.

(A) Human alveolar epithelial A549 cells (5×10^4 cells/200 µL) were cultured in serum-free DMEM (Wako Pure Chemical Industries, Osaka, Japan) and exposed to 62.5 mU/mL or 125 mU/mL hNE or 100 ng/mL LPS for 6 h. IL-8 concentration was analyzed by ELISA. (B) A549 cells (5×10^4 cells/200 µL) were cultured in DMEM supplemented with 10% fetal bovine serum (Japan Bio Serum Co. Ltd., Hiroshima, Japan) and exposed to various concentrations (500–5000 mU/mL) of hNE for 6 h. IL-8 concentration was analyzed by ELISA. Data represent the mean ± SD of quadruplicate experiments and were evaluated using one-way analysis of variance with Dunnett's multiple-comparisons test. *Significantly different from the control group at $P < 0.05$. Ctrl, control; hNE, human neutrophil elastase; SD, standard deviation.

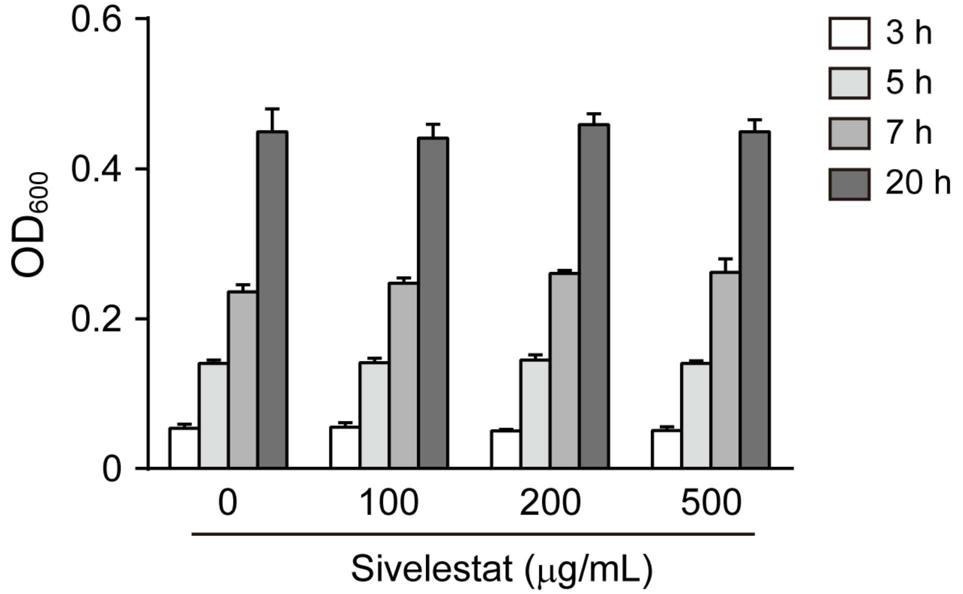

Figure S5. Sivelestat does not inhibit the growth of *S. pneumoniae* D39

S. pneumoniae D39 was inoculated into tryptic soy broth and cultured in the presence of various concentrations (100–500 μg/mL) of sivelestat for 3, 5, 7, or 20 h at 37°C. The OD of each test tube was measured at 600 nm. Data represent the means ± SD of triplicate experiments. OD, optical density; SD, standard deviation.

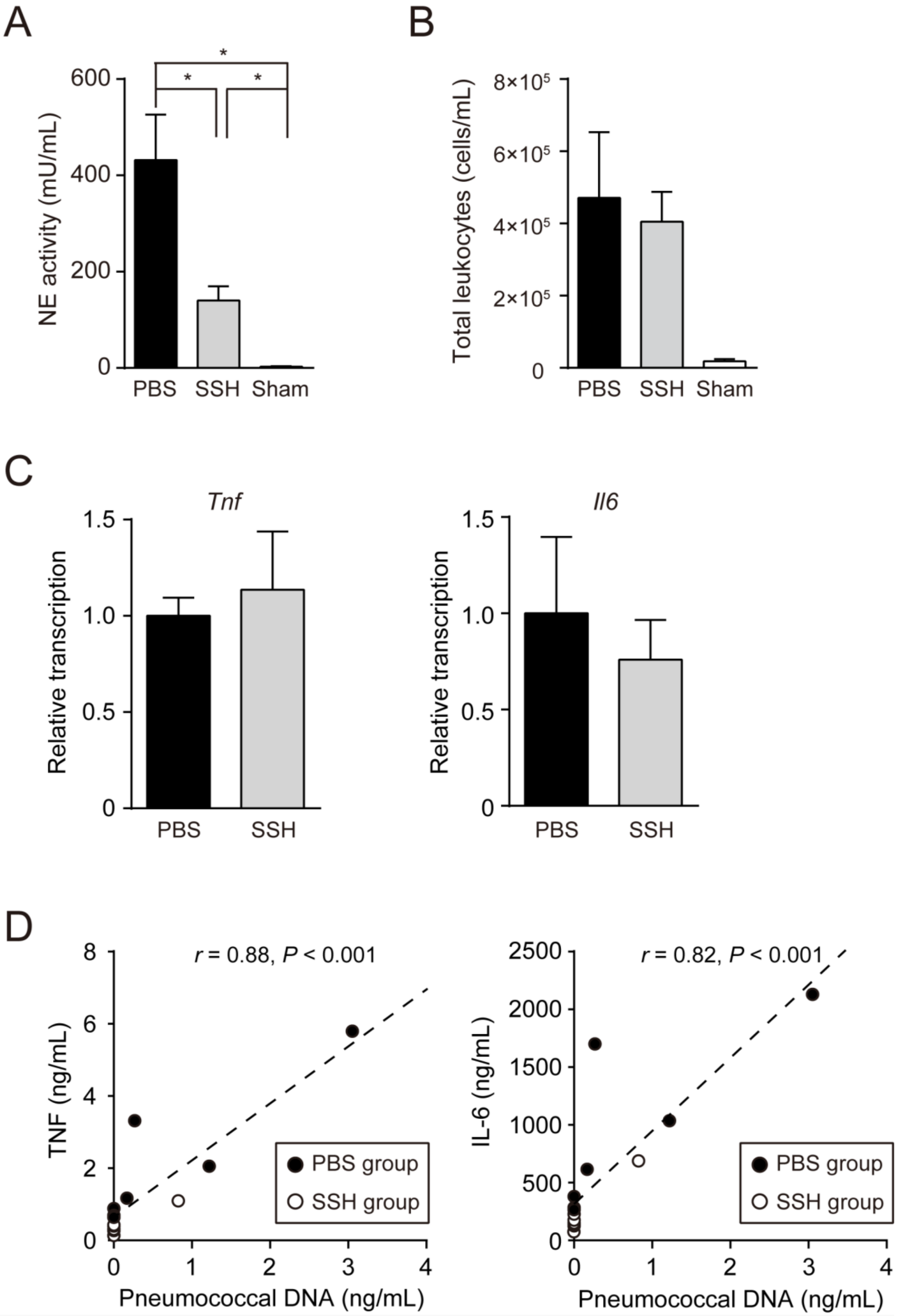
A
600
400
200
0
NE activity (mU/mL)
*
*
*
PBS
SSH
Sham
B
8×10^5
6×10^5
4×10^5
2×10^5
0
Total leukocytes (cells/mL)
PBS
SSH
Sham
C
Tnf
Il6
1.5
1.0
0.5
0
Relative transcription
PBS
SSH
D
r = 0.88, P < 0.001
r = 0.82, P < 0.001
8
6
4
2
0
TNF (ng/mL)
2500
2000
1500
1000
500
0
IL-6 (ng/mL)
PBS group
SSH group
0
1
2
3
4
Pneumococcal DNA (ng/mL)

Figure S6. Administration of an NE inhibitor decreases NE activity in BALF and ameliorates host innate immune responses *in vivo*

BALB/c mice (seven mice each) were intratracheally infected with *S. pneumoniae* D39 (2×10^8 CFU in 50 μL PBS). Unchallenged naïve mice (Sham group) were administered PBS only. NE inhibitor (SSH group; 50 mg/kg) or PBS (PBS group) was administrated intraperitoneally to the infected mice every 6 h. (A) NE activity in BALF was determined by a method using the NE specific substrate *N*-methoxysuccinyl-Ala-Ala-Pro-Val *p*-nitroanilide. (B) Total leukocytes in BALF were counted using Turk' s solution (Nacalai Tesque). (C) Real-time PCR was performed to quantify *TNF* and *IL6* mRNA in the lung tissue of mice. Data represent the means ± SD of quadruplicate experiments and were evaluated using (A, B) one-way analysis of variance with Tukey's multiple-comparisons test or (C) unpaired *t* tests. *Significantly different between indicated groups at $P < 0.05$. (D) Relationships between the cytokine level and pneumococcal DNA concentration in serum were assessed using the Pearson correlation coefficient. BALF, bronchoalveolar lavage fluid; CFU, colony forming unit; NE, neutrophil elastase; PBS, phosphate buffered saline; *r*, Spearman correlation; SD, standard deviation; SSH, sivelestat sodium hydrate.
